# Interventions that may increase control at the end of life in persons with dementia: the cross-cultural CONT-END acceptability study protocol and pilot-testing

**DOI:** 10.1186/s12904-023-01249-7

**Published:** 2023-09-27

**Authors:** Hanneke J.A. Smaling, Xu Jingyuan, Miharu Nakanishi, Shiri Shinan-Altman, David R. Mehr, Lukas Radbruch, Jan Gaertner, Perla Werner, Wilco P. Achterberg, Jenny T. van der Steen

**Affiliations:** 1 Department of Public Health and Primary Care, Leiden University Medical Center, Hippocratespad 21, Gebouw 3, Postal zone V0-P, P.O. Box 9600, Leiden, 2300 RC The Netherlands; 2 University Network for the Care sector Zuid-Holland, Leiden University Medical Center, Leiden, The Netherlands; 3 Department of Psychiatric Nursing, Tohoku University Graduate School of Medicine, Sendai, Japan; 4 School of Social Work, Bar Ilan University, Ramat Gan, Israel; 5 Department of Family and Community Medicine, University of Missouri, Columbia, MO USA; 6 Department of Palliative Medicine, University Hospital Bonn, Bonn, Germany; 7 Centre for Palliative Medicine, Helios Hospital Bonn/Rhein-Sieg, Bonn, Germany; 8 Palliative Care Center Hildegard, Basel, Switzerland; 9 Faculty of Medicine, University of Basel, Basel, Switzerland; 10 Department of Community Mental Health, University of Haifa, Haifa, Israel; 11 Center for Old Age Medicine, Leiden University Medical Center, Leiden, The Netherlands; 12 Department of Primary and Community Care, Radboud university medical center, Nijmegen, The Netherlands; 13 Radboudumc Alzheimer Center, Nijmegen, The Netherlands

**Keywords:** Palliative care, Dementia, End of life, Euthanasia, Assisted death, Advance care planning, Technology

## Abstract

**Background:**

Interventions such as advance care planning (ACP), technology, or access to euthanasia may increase the sense of control over the end of life. In people with advanced dementia, the loss of cognitive and physical function limits the ability to control care. To date, little is known about the acceptability of these interventions from the perspective of persons with dementia and others involved. This study will examine the cross-cultural acceptability, and factors associated with acceptability, of four end-of-life interventions in dementia which contain an element of striving for control. Also, we report on the development and pilot testing of animation video vignettes that explain the interventions in a standardized manner.

**Methods:**

Cross-sectional mixed-methods vignette study. We assess acceptability of two ACP approaches, technology use at the end of life and euthanasia in persons with dementia, their family caregivers and physicians in six countries (Netherlands, Japan, Israel, USA, Germany, Switzerland). We aim to include 80 participants per country, 50 physicians, 15 persons with dementia, and 15 family caregivers. After viewing each animation video, participants are interviewed about acceptability of the intervention. We will examine differences in acceptability between group and country and explore other potentially associated factors including variables indicating life view, personality, view on dementia and demographics. In the pilot study, participants commented on the understandability and clarity of the vignettes and instruments. Based on their feedback, the scripts of the animation videos were clarified, simplified and adapted to being less slanted in a specific direction.

**Discussion:**

In the pilot study, the persons with dementia, their family caregivers and other older adults found the adapted animation videos and instruments understandable, acceptable, feasible, and not burdensome. The CONT-END acceptability study will provide insight into cross-cultural acceptability of interventions in dementia care from the perspective of important stakeholders. This can help to better align interventions with preferences. The study will also result in a more fundamental understanding as to how and when having control at the end of life in dementia is perceived as beneficial or perhaps harmful.

**Trial registration:**

The CONT-END acceptability study was originally registered at the Netherlands Trial Register (NL7985) at 31 August, 2019, and can be found on the International Clinical Trials Registry Platform.

## Background

The central theme in what is regarded as a good death in western countries, is control [1]. Attempts to exert control at the end of life are emotionally charged and controversial. This is obvious from controversies around interventions such as euthanasia and assisted dying. In dementia, retaining control is inherently difficult due to cognitive decline. Issues with end-of-life care may be complicated by impaired decisional capacity and decreased ability to express preferences or complaints such as pain.

Palliative care assumes that anticipating and preparing for the end of life is beneficial [2, 3] and advance care planning (ACP) for the end of life is part of it. ACP involves discussing and documenting the desired direction of the patient’s care with patients and their family caregivers, thereby providing some control over current and future care. Positive effects of ACP on end-of-life care with dementia have been reported [4, 5]. However, dementia decreases the ability to imagine future situations, and coping (well) with dementia implies that preferences can change substantially [6]. For example, people’s prior perceptions of whether they will be enjoying life with moderate or severe dementia can be inaccurate. Also, the course of the disease cannot be predicted accurately [3]. If the perceived control turns out to be unrealistic or does not match desired levels or negatively impact relationships, perceived control can cause more distress than the perception of no control at all [7]. So, ACP in dementia is not without its challenges.

Despite many barriers to initiating ACP with persons with dementia [8, 9], some work indicates that ACP is acceptable in dementia care [5]. However, much less is known about whether persons living with dementia prefer a specific approach to ACP. For example, people might prefer to detail specific future treatments or they might simply prefer to discuss their goals and values regarding future care. In this study, we focus on the acceptability of two ACP approaches when applied to dementia care [10, 11]. The first ACP approach focuses on advance treatment orders in detail and thus capitalises on being in control. The second ACP approach focuses on setting global goals of care and coping with disease.

While ACP may provide some control over future decisions, there are interventions at the end of life that may provide more sense of control. At one extreme, people choosing assisted dying or euthanasia can dictate the time, place and manner of their death. Since 2002, Dutch law has regulated euthanasia with the Termination of Life on Request and Assisted Suicide Act, which legalized the ending of life by physicians at the request of patients suffering unbearably without hope of relief. The same year, Belgium adopted a law on euthanasia that is largely similar to the Dutch law [12]. Canada has legalized suicide assistance or euthanasia by physicians or nurse practitioners in 2016 [13, 14]. Euthanasia can also be legally practiced in Luxembourg, Colombia, Spain, some states of Australia, and in New Zealand. Physician-assisted suicide (PAS), not euthanasia, is legal in Switzerland, in a handful of US states and, more recently, in Austria. In Germany, the Federal Constitutional Court has overturned the prohibition of ‘business-like’ suicide assistance in the penal code in 2020, making it legally available again to commit suicide with the voluntary help of third parties [15].

The debate on legalizing euthanasia and PAS is controversial. Complete consensus seems to be unachievable due to incompatible normative frameworks that clash [16]. Differences in attitudes towards ending life upon request have been associated with differences in countries’ economic, religious and health-related factors [17]. Based on a 48-country survey study, there appears a rough West-East division in euthanasia acceptance among the European public, with relatively high acceptance in Western European countries compared to low to moderate acceptance in the other countries [18]. Public support for euthanasia and PAS in the US ranges between 47 and 69%, with higher public support for euthanasia than PAS [19]. The Netherlands is a country among the top in Europe with regard to public euthanasia acceptance [18, 19]. A total of 60% of the Dutch public agree euthanasia is acceptable for people with advanced dementia. Interestingly, far fewer physicians consider performing euthanasia in this population acceptable, only 8–24% [20]. In Japan, views about the acceptance of euthanasia, in general, are vary greatly depending on the study population [21–24]. So far, however, research about the acceptability of euthanasia across all these nations has primarily focussed euthanasia in general and on family caregivers, ‘the public’ or physicians. Much less is known about how acceptable people with dementia find euthanasia.

Technological advances in medicine imply patients, family caregivers and physicians need to make increasingly difficult end-of-life decisions. The growing use of technology in healthcare raises new questions such as, if, when and how the use of technological aids can affect the dying process. For example, distressing symptoms are common at the end of life in persons with dementia [25]. Patients’ self-report of discomfort is considered the “gold standard” [26]. However, in the terminal phase, patients may not be able to self-report their symptoms. This implies a loss of control in expressing their discomfort and needs and having to rely on others to observe discomfort. Automated pain and discomfort detection by continuous monitoring of facial expressions, activity and vital signs is being developed and may also be available for the dying [27, 28]. Nurses may then respond to technical signals from a distance rather than using bedside observations alone. As such devices are developed and implemented in care, it is important to understand the views of persons with dementia, their family caregivers and physicians about the use of monitoring technology (e.g. medical devices that can recognize distress) at the end of life.

To optimize care for persons with dementia, physicians need to understand differing attitudes toward end-of-life care and which factors to consider when discussing end-of-life decisions with persons with dementia and families from diverse backgrounds. Acceptability of interventions that increase control may vary by life view and personality. Control may be helpful in some cases. Early research in psychology for example, found that beliefs about having (some) control promote adjustment with life-threatening disease [29, 30]. With uncontrollable disease, however, acceptance in the sense of ability to tolerate the nature of the disease can help redirect personal goals, change personality in a positive way and strengthen relationships [31]. Control or goal engagement is the opposite, in a way, to acceptance or letting go. However, it is more complicated than that. For example with aging, people adjust goals to what is feasible which buffers their sense of control [32]. Moreover, both control and acceptance can improve wellbeing. Also, manageability, together with comprehensibility and meaningfulness allows people to better cope with adversities [33, 34]. More generally, there is a need for research to address the complexities and underlying question of how and when attempts at retaining control over the end of life is beneficial and acceptable for whom, and to explain individual and possible cross-national differences.

In this study, the perspectives of persons with dementia, their family caregivers, and physicians in six countries (Netherlands, Japan, Israel, US, Germany, Switzerland) will provide insight into the cross-cultural acceptability in dementia care of two ACP approaches, the use of monitoring technology at the end of life, and euthanasia.

### Objectives

The primary objective is to investigate the acceptability of the four interventions (i.e., whether the participants would want the interventions at the end of life: persons with dementia for themselves, family caregivers for their relative, and whether physicians would use it at request). The secondary objectives include examining differences in acceptability between groups and countries, and to explore associations between acceptability and other participant characteristics (e.g., demographics, variables indicating life view such as religion, and personality variables such as coping style). We use open-ended questions in the interviews to examine qualitatively possible ambiguity regarding being in control through the interventions and in what situations and why the participants feel the interventions are or are not acceptable.

## Methods

The Attempts to CONTrol the END of life in people with dementia (CONT-END) acceptability study was originally registered at the Netherlands Trial Register (NL7985) at 31 August, 2019, and can be found on the International Clinical Trials Registry Platform. The study has a cross-sectional mixed-methods design with participants evaluating intervention vignettes and completing a survey. The vignette scripts, storyboards and early versions of the animation videos were pilot-tested between March and June 2020. Data for the CONT-END acceptability study are collected in the Netherlands, US, Germany, Switzerland, Japan and Israel. The study will be conducted according to the principles of the Declaration of Helsinki (Fortaleza, Brazil, October 2013).

An independent Ethics Advisory Committee was installed as a requirement of the funder, which included representatives from most participating countries in the study, including one member of the Alzheimer Association, to ensure that ethical issues that may arise are also treated in a manner that is sensitive to the international differences. The main remit is to review documents and to monitor the project’s progress from an ethics point of view. The Ethics Advisory Committee members do not receive compensation for their time and are not otherwise involved in the study.

### Selection of countries

The interventions offer different levels of control and their acceptability may vary between countries, groups and individuals. The countries in this study were selected based on literature about large differences in end-of-life care, access to means to exercise control and norms regarding autonomy, technology and sanctity of life [35–45].

First, there may be differences in acceptability of the ACP interventions between countries. This is based on literature about patient autonomy versus relational autonomy or influence of the family or professional caregiver on end-of-life decision making, and legalisation of assisted suicide and not euthanasia in Switzerland and US states (e.g., [35]). Physicians in Germany have much decision-making authority [45]. Physicians in the Netherland are more “paternalistic” than in the US regarding end-of-life treatment decisions in dementia [46]. However, in the Netherlands there is also a focus on self-management with health even proposedly defined as “the ability to adapt and to self-manage” [47].

Second, inhabitants of technology-minded countries with aggressive life-prolonging treatment and infrequent withholding of treatment at the end of life to allow a “natural course” might be most in favour for using technology in the dying. By contrast, in the Netherlands, less aggressive care is common in dementia at the end of life (e.g. low antibiotic use and invasive rehydration) [48–50], as is withholding life-prolonging treatment if it does not improve quality of life [51]. Rates of withholding treatment may be comparable in Switzerland [43, 52]. Further, three countries are known for technological advances: Israel, Japan and the US. These countries are also known for high rates of feeding tubes in people with advanced dementia [36, 38, 39].

Third, a number of publications [36, 44] have shown that sanctity of life is an important value in end-of-life decision making in Israel and Japan. Germany is distinct within Europe for low acceptance of euthanasia [45]. In the Netherlands, on the other hand, euthanasia is legal when fulfilling certain criteria, and the country was at the forefront of euthanasia legislation [41, 45].

### Hypotheses

For the acceptability of the interventions per group, we expect that the ACP intervention with detailed advance treatment orders will be most often acceptable and euthanasia least often acceptable in physicians. No hypotheses on acceptability rated by persons with dementia compared to the other groups are formulated because not enough is known about their views on this matter to formulate specific hypotheses. With regard to acceptability by country, we hypothesize that the ACP intervention with detailed advance treatment orders will be most often acceptable in countries where patients have high autonomy in decision making about medical procedures and care, and where people may feel they need a defence against medical overtreatment, while sanctity of life is not necessarily a dominant principle (in particular, the US). Technology for symptom monitoring when unable to self-report, such as use of cameras in the dying phase, is expected to be most often acceptable in technology-minded countries (Japan, Israel and US). Euthanasia may be most often acceptable in countries that have euthanasia or PAS regulation already in place for a while (in particular the Netherlands and Switzerland) and the least acceptable in countries where ending life is highly controversial (Germany, Israel and Japan).

For the explorative analyses, we expect demographics, variables indicating life view such as religion, view on dementia, attitudes regarding life-prolonging treatment and death and dying, and variables indicating personality such as coping strategy to be associated with acceptability of the interventions. These aspects of life view and personality are selected because they may relate to control. In explaining diversity in acceptability of ACP, individual differences may be more important than differences between countries and respondent groups (hypothesized based on a study in physicians in the UK and the Netherlands; [53]), with those tending to planning as a coping strategy more likely to find the interventions acceptable. Contrary, it could be that people with higher death anxiety are less likely to find the interventions acceptable, as it has been related to fewer preparations for death in people with advanced cancer [54, 55].

### Participants

We initially aimed to include a total of 900 participants from six countries (Netherlands, US, Japan, Israel, Germany, Switzerland), comprising 300 persons with (early) dementia, 300 family caregivers and 300 physicians. From each country, we planned to recruit 50 dyads of persons with dementia and their family caregivers, and 50 physicians. Recruitment procedures for persons with dementia, their family caregivers and physicians are adapted for each participating country in close collaboration with our local partners. We amended the participant target due to difficulty recruiting persons with dementia and family caregivers (see paragraph ‘Adjustments to protocol’).

We work with healthcare professionals, primarily physicians working in memory clinics and hospitals, to recruit dyads of a person with dementia and their family caregiver. We also recruit via general practitioners (GPs) and dementia case managers. When persons with dementia visit their physician, the physician will offer an information package to eligible participants. Participants can then: (a) directly contact the research team, or (b) inform their physician about their decision. The physician will then ask for permission to forward the contact information of the pair to the research team.

For the recruitment of the physicians, the study is announced via relevant webpages and newsletters of local professional networks and educational institutions. The researchers also directly contact physicians who are already in their professional network by email to inform them about the study. The researchers send physicians who are interested an information package about the study and call them to explain the study in more depth and to schedule an appointment for the assessment.

The inclusion criteria for the person with dementia are: (1) has a diagnosis of irreversible dementia established by a physician; (2) has been informed about and is aware of their diagnosis; (3) has a family caregiver who is willing to participate in the study; (4) has decision-making capacity and is able to communicate through sufficient memory and language; (5) is able to understand and sign the consent form; (6) has adequate vision and hearing (can be achieved by using corrective lenses and hearing aid if required); and (7) consents to participate.

There is no formal capacity test to avoid feelings of failure if that would result in denying participation. We rely on the clinician’s estimation of whether people have capacity to decide to participate in the study and are able to do so. If needed, we provide the referring clinicians with three relevant items of the Dementia Severity Rating Scale to aid clinical judgement of the capacity to give meaningful responses in the interview [56]. For the ‘Memory’ item a score of ≤ 2, ‘Speech and Language’ item a score of ≤ 3, and ‘Ability to make decisions’ item a score of ≤ 2 indicate sufficient ability for the purpose of this study. If the person with dementia is currently affected by a severe psychiatric disorder (e.g., major depression, schizophrenia, substance abuse, post-traumatic stress disorder) as diagnosed by a psychiatrist, psychologist, or physician, or if the participant is expected to die in a few weeks, they are excluded.

Family caregivers can participate if (1) they are willing and able to participate in the study; (2) the person with dementia they care for is willing and able to participate; (3) are ≥ 18 years old. Physicians can participate if they practice a specialty that includes provision of end-of-life care for persons with dementia. Depending on the country, the specialty could be primary care physicians such as GPs, elderly care physicians, geriatricians, geriatric psychiatrists, neurologists, and palliative care physicians.

### Study setting

The study setting differs for the person with dementia and their family caregiver versus physicians. Ideally, the assessment with the person with dementia and their family caregiver takes place at the local research site, at their home or the practice of their healthcare professional. This can be at a memory clinic, hospital, or their physician’s practice. The assessment with the physicians will take place at their office or online via videocall.

### Procedure

Participation in the study consists of a one-time assessment of 60 (physicians) to 90 min (person with dementia and family caregiver). During the assessment, the participants view four animation videos (range duration 1:53 to 4:51 min depending on the intervention and language). After each video, they are interviewed. The order of the two ACP-videos and the Technology video is randomized by an independent statistician. Due to the potential sensitivity of the topic, the Euthanasia video is shown last. The participants also complete a brief survey with items about attitudes concerning the end of life, views on dementia, decision-making, coping, and demographic information. Table 1 gives an overview of all the instruments and items in the survey. If needed, a translator is present during the assessment.

The in-person assessment with the person with dementia and their family caregiver is be conducted by two researchers or one researcher and a volunteer. Volunteers and translators are trained by the research team in advance. After finishing the assessment, the pair is debriefed and receives information about what to do if they feel distressed afterwards related to the assessment.

**Table 1:** Overview of the instruments per group

Constructs	Items in the survey	Respondent group		
		Person with dementia	Family caregiver	Physician
Attitudes towards the end of life	• 4 items from the Death Anxiety Questionnaire (58)	X	X	X
	• one item of each of the five subscales from the Death Attitude Profile-Revised (DAP-R) (59, 60)	X	X	X
	• Item about general level of comfort with talking about the end of life (11)	X	X	X
Caregiver burden	Zarit Burden Interview, 6-item version (ZBI-6) (61–65)		X	
Concerns about future	Three items from the subscale ‘Preparation for end of life’ of the Quality of life at the end of life (QUAL-E) (68)	X		
Coping styles	Brief COPE subscales: Use of instrumental social support; Active coping; Denial; Use of emotional social support; Acceptance; Planning; Religious coping (69, 70)	X	X	X
Decision-making	• Is your relative capable of making decisions on medical treatments by themselves? (11)		X	
	• Does your relative’s faith or spiritual background influence decisions about care and treatment? (11, 71)		X	
	• Medical decision making (11)			X
Dementia	• Dementia diagnosis • Quick Dementia Rating System (QDSR) - cognitive subscale (72)	O O		
Demographic variables	Age, gender, educational level, country of birth, religious background, worked in healthcare, relation to person with dementia, living situation of person with dementia, work status family caregiver	O	X	X
Goals of care	• The most important goal of [your relative’s] health care is to preserve my [his/her] life as long as possible, even if that requires treatments that may cause pain or discomfort. (11)	X	X	
	• When you think about the goals of care for your relative, which of the following do you most strongly consider? what you think your relative would probably want for themselves, what you want for your relative, what you think the physician of your relative wants for themselves, and don’t know		X	
Illness cognition	Rating of the following statements:			
	• Dementia is a disease you can die from. (11, 37)	X	X	X
	• Dementia is a normal part of the ageing process. (73, 74)	X	X	X
	• Palliative care applies from the time of diagnosis to the stage of severe dementia. (75)			X
Illness perception	Brief Illness Perception Questionnaire (IPQ-B) - one item from the Consequence, Personal Control, Illness concern and Emotional representation dimensions (76–78)	X		
Interpersonal closeness	Inclusion of other in the self scale (IOS) (79)		X	
Locus of control	4-item version of the Locus of Control scale (IE-4) (80)	X	X	X
Mood	2-item Patient Health Questionnaire depression module (PHQ-2) (81)	X O	X	
Personal experiences with dementia	Have you personally experienced a family member or friend having advanced dementia at the end of their life? (82)	X	X	X
Preparedness	Item about preparedness for the end of life of relative (83)		X	
Work experience	Specialty, additional palliative training, work setting, years of experience with dementia care (82)			X

*Note*: X = fills in questions about themselves, O = questions about person with dementia filled in by family caregiver

### Sample size calculation

The number of participants is determined by the primary objective of the trial (acceptability). We will use logistic regression analyses and before the COVID-pandemic, we aimed at a total of 6 (countries) x 3 groups) x 50 = 900 respondents for sufficient power according to the 10 events per variable rule of thumb, for a minimum acceptability or non-acceptability rate of 10%. Therefore, with percentages of 10% and up, power would suffice to assess differences between 6 countries and 3 respondent groups. Testing of associations with other characteristics, i.e. life view and demographic and personality characteristics, is exploratory. Also see paragraph ‘Adjustments to protocol’.

### Adjustments to protocol

Due to COVID-19 hitting the world in the beginning of 2020, the start of the data collection was postponed to July 2020 and is extended till 2023.The COVID restrictions caused severe delays in the data collection. Healthcare providers were hesitant to join the study both as a participant and to assist in the recruitment of persons with dementia and their family caregivers due to the increased workload and wanting to protect their patients. Therefore, adjustments were made to the protocol. The recruitment strategies for persons with dementia and family caregivers were expanded to recruiting through dementia cafes and social media. In the US, an online service of patients who have volunteered to participate in research studies (Research Match) and electronic resources were used to identify additional persons with dementia. When identified from the electronic record, their physician was asked if it is acceptable to contact the patients and caregivers. A telephone screening script was developed to check whether the person with dementia and family caregiver met our inclusion criteria.

After completing the data collection of the Dutch and US physicians, and after interviewing 15 dyads of person with dementia and family caregiver in the Netherlands, we decided to reduce the number of interviews to 15 per country per group. This was because 50 dyads was no longer considered feasible within the remaining study time and because we found data saturation in the Dutch data was reached after 12–15 interviews [57]. For physicians, we decided to offer a full self-report online version of the assessment after 15 interviews, where they can view the animation videos themselves and then indicate whether they find each intervention acceptable. Open text fields are provided for an optional explanation of the answers. In general, this online option was not deemed feasible for the dyads (e.g., limitations in digital-literacy, availability of technology for online meeting, etc.), and not approved of by the Dutch ethics committee due to the potential sensitive topics. For the dyads, the focus will thus be on the qualitative data.

For the adjusted protocol a new power calculation was performed. We will use logistic regression analyses and expect that collecting physician data (n = 50) in a total of 6 (countries) results in 300 physician respondents. Power depends strongly on the type of intervention as this has varied, so far in data collected in the Netherlands, the US and Japan, from acceptable for almost all (advance care planning interventions) to acceptable for closely around half. According to the 10 events per variable rule of thumb, for 7 variables, with 300 respondents, 70 events suffice for acceptability in ratios close to half of respondents, but would not suffice for interventions that are (not) acceptable for the large majority of the respondents. However, examining associations with acceptability is less relevant for interventions which are endorsed or rejected by the great majority.

### Primary study parameter(s)

The primary outcome is acceptability of the interventions, assessed during the interview. The two primary acceptability measures are: [1] whether the participant finds the intervention acceptable for dementia care, and [2] whether they want the intervention for themselves. This means, whether persons with dementia want it for themselves, family caregivers for their relative and whether physicians would use it at request. The primary outcome is a categorical variable with three categories (‘yes’, ‘no’ and ‘don’t know’ as a valid answering option).

### Secondary study parameters

The secondary outcomes will be assessed using the survey. Table 1 provides an overview of the instruments per group. When not available, the forward-backward translation technique was used to translate the instruments in all languages necessary for the current study (i.e. Dutch, English, German, Japanese and Hebrew). The instruments were translated to each new language by two native speaking researchers independently. They compared their translations and came to a consensus, followed by back-translation by a professional, independent translator. The translation was reviewed by the two researchers and discussed with a third person, the principal investigator or coordinating local partner. The professional translator and original authors were consulted for any remaining specific questions.

To assess attitudes towards the end of life, we use the death anxiety questionnaire [58] as an indicator of a general or global fear of death as a whole. The response options are *strongly disagree* [1], *disagree* [2], *agree* [3] and *strongly agree* [4], and we added *I don’t know* as an additional option. A sum-score can be calculated by summing the scores of the items with a high score reflecting more anxiety about dying. Also, we selected one item of each of the five subscales of the Death Attitude Profile-Revised (DAP-R) [59, 60] to assess attitudes towards death. including: ‘*The prospect of my own death arouses anxiety in me*’ (Fear of death), ‘*I avoid thinking about death altogether*’ (Death Avoidance), ‘*Death is neither good nor bad*’ (Neutral Acceptance), ‘*I look forward to a life after death*’ (Approach Acceptance), and ‘*I see death as a relief from the burden of this life*’ (Escape Acceptance). We will look at the individual items. The seven response categories were reduced to five to match those of the death anxiety questionnaire. Additionally, we use a single item asking the participants about their general level of comfort with talking about the end of life with the response options *very comfortable*, *somewhat comfortable*, *somewhat uncomfortable*, *very uncomfortable*, *don’t know* [11].

To assess caregiver burden, we use the shortened 6-item version of Zarit’s well-tested caregiver burden interview (ZBI-6) [61–65]. Items are scored on a five-point scale, with a cut-off score of ≥ 13 considered as a clinically significant burden [66]. The items are added up to create a sum-score.

The three items from the subscale ‘Preparation for end of life’ from the Quality at the end of life in terminal patients (QUAL-E) [67, 68] are used to indicate concerns of the person with dementia about the future after they have died. The items are scored on a 5-point Likert scale ranging from *not at all* to *completely*. The items are added up to create a subscale score.

To assess coping strategies in all three groups, we use the Brief COPE subscales Active coping, Planning, Using instrumental support, Using emotional support, Denial, Acceptance, and Religion [69, 70]. Each subscale contains two items that are scored from 1 (*not doing it at all*) to 4 (*doing it a lot*). The items are added up to create the subscale score.

We will use two items about medical decision making. The family caregiver is asked whether their relative is capable of taking decisions on medical treatments by themselves using the response option *yes*, *sometimes or in part*, *no* and *I don’t know* [11]. Next, they are asked to indicate whether their relative’s faith or spiritual background influences decisions about care and treatment using the categories *yes strongly*, *yes somewhat*, *no*, *I don’t know* [11, 71]. The physicians are asked to rate the item *When you think about the goals of future care for your patients with dementia, which of the following do you most strongly consider*? Using the response options *what you think your patient would probably want for themselves*, *what the family/loved one of the patient wants*, *what you as the physician of your patient wants for them* and *don’t know* [11]. We will look at the individual items.

The family caregiver is asked about the type of dementia and the month and year of the diagnosis. As an indicator of the stage of dementia, we will ask the family caregiver to complete the cognitive subscale of the Quick Dementia Rating System (QDRS) [72]. This subscale corresponds to the Mini Mental State Examination classifications (28–30, 24–27, 18–23, 10–17 and 0–9)). The cognitive subscale consist of 4 domains: memory and recall, orientation, decision making & problem solving, and language & communication abilities. Each domain has five possible answers, with higher numbers reflecting more severe cognitive symptoms. The items of QDRS are added up to create a sum-score.

The family caregiver and person with dementia are asked to indicate the extent to which they (dis)agree with the following statement: *The most important goal of [your relative’s] health care is to preserve my [his/her] life as long as possible, even if that requires treatments that may cause pain or discomfort* using a scale ranging from *strongly agree* [1] to *strongly disagree* [5]. *Don’t know* [9] is also a valid option [11]. The family caregiver is also asked to indicate which they most strongly consider when thinking about the goals of care for their relative using the categories *what you think your relative would probably want for themselves*, *what you want for your relative*, *what you think the physician of your relative wants for themselves*, and *don’t know*.

Illness cognition refers to the dementia. All three groups are asked to rate the extent they agree with two statements: *Dementia is a disease you can die from* [11, 37] and *Dementia is a normal part of the ageing process* [73, 74] using the response options *strongly agree* [1] to *strongly disagree* [5] and *Don’t know* [9]. Physicians will also be asked to rate the statement *Palliative care applies from the time of diagnosis to the stage of severe dementia* using similar response categories [75].

Illness perception also refers to the dementia. The person with dementia is asked to rate four selected items of Brief Illness Perception Questionnaire (IPQ-B) [76–78] that are relevant to our research questions. The four items consist of one item from the Consequence, Personal Control, Illness concern and Emotional representation dimensions. The items are scored using a 11-point (0–10) Likert-type scale with higher scores reflecting more negative perceptions. The items are added up to create a sum-score.

Inclusion of other in the self (IOS) was assessed using the IOS Scale [79]. This is a single-item, pictorial measure of relationship closeness. It consists of seven pairs of circles, one that includes the word *self* and one that includes the word *other*, that overlap to different degrees. Family caregivers are invited to select the pair that best describes their relationship with the person with dementia.

Locus of control (i.e., personal belief about whether outcomes of behaviour are determined by one’s actions or by forces outside one’s control) is measured using the 4-item locus of control scale (IE-4) [80]. The items are scored on a scale ranging from *doesn’t apply at all* [1] to *completely applies* [5]. Higher scores on Internal Locus of Control of Reinforcement (Item 1 and 2) relate to higher levels of internality (internal control), while higher scores on External Locus of Control of Reinforcement (Item 3 and 4) relate to higher externality (external control). Internal and External Locus of Control are calculated separately.

To assess possible depression in the person with dementia and their family caregiver, the 2-item Patient Health Questionnaire depression module (PHQ-2) [81] is used. The PHQ-2 asks about the frequency of depressed mood and anhedonia over the past 2 weeks, scoring each as *not at all* (0) to *nearly every day* [3]. The time frame was adjusted to one month, as we were interested in mood over a longer period of time. Additional to rating their own mood, the family caregiver is asked to rate the mood of their relative using these two items.

The item *Have you ever accompanied a member of your family or friend suffering from advanced dementia at the end of their life* is used to measure personal experience with dementia [82]. The item is scored using *yes* [1] or *no* (0).

The family caregiver is asked about their preparedness for the end of life of their relative using the item *If your relative were to die soon, how prepared would you be for their death* [83]. We adjusted the 3 response options to a 7-point scale ranging from 1 (*not prepared at all*) to 7 (*prepared as much as possible*) to allow for greater precision [84].

Demographic information includes age, gender, educational level, country of birth, religious background, worked in healthcare, relation to the person with dementia, living situation of the person with dementia, and work status of the family caregiver. The family caregiver provides the demographic information about the person with dementia. For physicians, we also ask details about their work experience; specialty, additional palliative training, work setting, years of experience with dementia care [82].

Finally, the members of the CONT-END acceptability study research team are asked to fill in a questionnaire about their own attitudes and views regarding the study and acceptability of the four interventions to increase transparency and their reflective awareness. This will be done at the beginning and after the data collection.

### Pilot testing of the instruments

The pilot testing of the items for the survey was conducted in the Netherlands. During a face-to-face visit, the survey for people with dementia was read out loud to one person with dementia (female). Her family caregiver (female) examined the survey for family caregivers. Six people aged over 65 years (five females, one male) received the digital version of the survey for family caregivers. One of them (female) also evaluated the survey for people with dementia digitally. The participants were asked to indicate whether the items were easy to understand and whether they were confronting. Most items were considered clear and not confronting. Items from validated questionnaires were not adjusted despite occasional comments from the participants. The time and effort needed to fill in the survey was also acceptable. Some participants commented that it was difficult to choose one option when they had a nuanced view. Minor adjustments were made in the instructions and response options (for example, changing “agree” and “disagree” if not the endpoint of the scale into “somewhat agree” and “somewhat disagree” for the questions on attitudes about the end of life).

### Development of the animation videos

With a professional media production group, Knowledge Media Research Centre, we developed video vignettes to explain each intervention in a standardized manner, aiming at a neutral stance, not slanted to either acceptance or non-acceptance. We choose animation videos to support understanding with visual and audio cues. Each video includes a brief introduction, an example and a summary of the main aspects of the intervention. To facilitate orientation, each video has its own colour. Further, a mnemonic for each video in the form of a hand-out with screenshots summarizes the content of the animation video for the persons with dementia and their family caregivers. The handout about euthanasia is supplemented with a sheet explaining local rules and explanation of differences with locally better-known PAS or palliative sedation.

The animation videos were developed iteratively navigating the following steps: step 1, developing the scripts; step 2, reviewing, pilot-testing and refining the scripts; step 3, reviewing, pilot-testing and refining the animation videos; step 4, translating and adapting the animation videos into other languages.

Step 1: Developing the scripts. The initial scripts of the animation videos were developed in English. The scripts were written with the goals in mind that they should be applicable in all six countries and understandable for all three groups of participants in this study.

Step 2: Reviewing, pilot-testing and refining the scripts. The initial scripts were reviewed by the CONT-END research team, consisting of an elderly care physician, GP, epidemiologist, communication expert, psychologists, and anthropologist. Next, the partners from the other countries were consulted on whether cultural adjustments were needed. This resulted in minor changes, for example, to replace “euthanasia” in the American and German context with “termination of life on demand” and “Aktive Sterbehilfe”. The independent international Ethics Advisory Committee reviewed the scripts as well. For the euthanasia video, they suggested emphasizing the hypothetical nature of the video to avoid misunderstanding in countries where active euthanasia is illegal. All feedback was discussed within the research team and adjustments were made to the scripts.

The scripts were then translated into Dutch following the forward-backward technique and read out loud during home visits to four pairs of persons with early dementia (three female, one male) and their family caregivers (three female, one male). The family caregivers were the partner or adult children of the persons with dementia. The participants were recruited through GPs. Participants were asked to comment on whether the scripts were easy to understand, neutral, and whether they were confronting. Based on the feedback from the participants, the scripts were thoroughly revised; the language and plot of the videos were simplified, overly positive words, such as “valuable”, were changed into neutral words and the difference between the two ACP interventions was clarified. The English scripts were adjusted accordingly.

Step 3: Reviewing, pilot-testing and refining the animation videos. Based on the updated scripts, Dutch and English animation videos were developed and produced by a team of experts from Knowledge Media Research Centre and a freelance animation artist. The Dutch animation videos were pilot-tested digitally due to COVID-19 related restrictive measures. Eight Dutch participants (five female, three male) with experience with dementia or an age above 65 viewed the animation videos. They were recruited through the Regional Older people Advisory Board connected to the Leiden University Medical Center (LUMC) and via the researchers’ network. In general, the participants found the videos clear and easy to follow. Some participants pointed out that the videos gave the impression that all discomfort could be detected by the technology, and that there were no obstacles for euthanasia once all criteria are met. Potentially confronting elements in the videos were also discussed, for example, the animation of an inflamed lung reminded some people of a COVID19-infection.

The English version of the euthanasia animation video was reviewed by the Ethics Advisory Committee. They suggested adding hand-outs and making the euthanasia procedure more clear for non-Dutch viewers. The English technology video was presented at the Alzheimer’s Association International Conference 2020 [85] to collect feedback from a professional audience. They deemed the animation video suitable. All feedback gathered from this step was discussed within the research team and yet more minor adjustments in scripts and animation videos were made.

Before and after the pilot-testing, readability tests were performed for the scripts with an online tool (available at: https://www.webfx.com/tools/read-able/check.php). The results suggested that almost all of the updated scripts (rated as grade 9 to 10) were more easily readable than the initial versions (grade 10 to 13). The updated script about technology had an average level of grade 9 and should be easily understood by 14 to 15 year-olds. The other three updated video scripts were rated as grade 10, which should be easily understood by 15 to 16 year-olds.

Step 4: Translating and adapting the animation videos into other languages. The English scripts were translated into Hebrew, German, and Japanese using the same forward-backward translation process as for the Dutch scripts. The translated scripts were reviewed by the local partners and at least two older adults (> 65 years) from each country to check whether anything in the scripts was too confrontational or difficult. (Cultural) adjustments in wordings were made when necessary. The Dutch research team and the local partners provided feedback on the timing of the animation videos and approved of the final versions.

### Data analyses

Descriptive statistics will be used to present the acceptability of the four interventions. The internal consistency of the scales will be calculated using Cronbach’s alpha. Descriptive statistics will be calculated to describe the demographics of the respondent groups per country.

We will use logistic regression analyses with acceptability of the intervention as the dependent variable to examine differences in acceptability between group and countries. ‘*Do not know*’ as a response option is often ignored and excluded from the denominator. A dichotomous variable will be create with a denominator of ‘acceptable versus not acceptable plus do not know’. The analyses will be unadjusted and adjusted for the most important life view or personality parameter (religion for euthanasia, planning for the other interventions). We will explore associations between acceptability and participant characteristics (gender, educational level, age, severity of cognitive impairment for persons with dementia, and palliative care training for physicians) and personality (locus of control, coping), depression, medical decision making, professional or personal experience with dementia, physician’s comfort with end-of-life conversations, and caregiver burden, and life view (religion, death anxiety/attitude, preparedness for end of life, priority quality vs. quantity of life, illness cognition). For this, we will use stepwise backward regression analyses. The analyses will be done with and without adjustment for country and group (forced in the stepwise regression analyses).

Qualitative data from the interviews will be used to map possible ambiguity regarding being in control through the interventions, and as to why and in what situation the participant feels the interventions are acceptable. A content analysis regarding acceptability of the interventions will be conducted. Additionally, across all interviews, we will conduct a thematic analysis on perceptions of control. Data collection and data analysis will be executed iteratively. Coding is supported by the software program ATLAS.ti.

### Data management and monitoring

To protect participant privacy, the data will receive a unique identification code with linkage keys to be stored securely separately from the data. Names and other information that could directly identify the participant are therefore omitted. As long as it is necessary to trace data to an individual participant, the participant identification code list per country will be maintained to enable linking the data to a particular participant. This will be until the data has been fully cleaned and no new cleaning issues have arisen.

Participants can choose to fill in the survey digitally or on paper. We will use a secure certified online program for the digital questionnaires, Castor (Amsterdam, The Netherlands). The data from the paper survey will be entered in the online program by the researcher. We will subject 10% of the data to a random audit by a second researcher to test the accuracy of quantitative data entry of the paper survey and forms. The audio files of the interviews will be deleted after they have been transcribed and the transcripts have been checked. Data will be entered and stored using a secure data server. To allow for cross-national comparison, the study will be performed in non-EU countries according to the same protocol that is acceptable for use in the EU countries.

### Dissemination

The findings will be submitted to relevant peer-reviewed scientific journals and national and international conferences within the field. We will share the results with key stakeholders involved in both homecare and long-term care of persons with dementia to help improve clinical practice. The animation videos are available from the PI upon reasonable request after conclusion of the data collection.

## Discussion

In this study examined the perspectives of persons with dementia, their family caregivers and physicians in six countries (Netherlands, Japan, Israel, US, Germany, Switzerland) to provide insight into the cross-cultural acceptability in dementia care of two ACP approaches, the use of monitoring technology at the end of life, and euthanasia. We anticipated and experienced practical and operational issues in conducting the study. First, due to COVID-19 restrictive measures, more assessments with physicians will be conducted online than initially anticipated. The online assessment offers more flexibility, possibly lowering the threshold for physicians to participate in the study and allowing the research teams to collect data simultaneously in multiple countries. While the COVID-19 pandemic may make it difficult to find physicians who have time to participate in a study, it also highlights the importance of timely ACP and monitoring distress signals at the end of life, possibly motivating physicians to share their opinions about these interventions. Second, we strive to conduct the assessments with the person with dementia and their family caregiver in person. It could be that the person and family caregiver are reluctant to receive visitors, even if allowed. The online option with these groups will also be offered as a last resort option after consultation with the local institutional review board and based on the judgement of the family caregiver if this would be a feasible option for their relative. Third, the recruitment of persons with dementia matching our inclusion criteria may be challenging in countries where dementia is generally diagnosed in a late stage of the disease.

The study limits professional caregivers’ perceptions to those of physicians and nurse practitioners with medical responsibilities whereas nurses have a role in providing these interventions as well. Further, data will not be collected simultaneously in all countries and groups; as a result of the sequential cross-sectional design, we cannot adjust for possible trends over time.

Strengths of this study include the cross-cultural aspect, comparing the perspectives of persons with dementia, family caregivers, and physicians, the sample size, mixed-method design, and initiative to increase the researchers’ transparency and their reflective awareness. The animation videos were developed using input of international experts, have been pilot-tested, and have been reviewed and approved by an independent Ethics Advisory Committee. This study will contribute to a more fundamental understanding of how acceptable a range of interventions differing in level of control offered is, and more generally, as to how and when having control at the end of life in dementia is perceived as beneficial. This facilitates aligning interventions with preferences and will help improve dementia care.

## Data Availability

The data that support the findings of the pilot study are available upon reasonable request and with permission of the principal investigator: Jenny van der Steen PhD, email jtvandersteen@lumc.nl.

## Declarations

The protocol of the pilot study has been reviewed by the Medical Ethics Committee - Leiden The Hague Delft (METC LLD), and they declared it to be exempt from the Medical Research Involving Human Subjects Act (WMO), protocol number: N19.098. Informed consent will be obtained from all participants.

## Abbreviations

ACP: advance care planning
DAP-R: Death Attitude Profile-Revised
ERC: European Research Council
EU: European Union
GP: general practitioner
IE-4: 4-item locus of control scale
IOS: Inclusion of other in the self scale
IPQ-B: Brief Illness Perception Questionnaire
LUMC: Leiden University Medical Center
PAS: physician-assisted suicide
PHQ-2: 2-item Patient Health Questionnaire depression module
QDRS: Quick Dementia Rating System
QUAL-E: Quality at the end of life in terminal patients
US: United States
ZBI-6: Zarit’s caregiver burden interview

## References

[1] Cottrell L, Duggleby W (2016). The good death: an integrative literature review. Palliat Support Care.

[2] Radbruch L, De Lima L, Knaul F, Wenk R, Ali Z, Bhatnaghar S (2020). Redefining palliative care—A new consensus-based definition. J Pain Symptom Manag.

[3] van der Steen JT, Radbruch L, Hertogh CM, de Boer ME, Hughes JC, Larkin P (2014). White paper defining optimal palliative care in older people with dementia: a Delphi study and recommendations from the European Association for Palliative Care. Palliat Med.

[4] Dixon J, Karagiannidou M, Knapp M (2018). The effectiveness of advance care planning in improving end-of-life outcomes for people with dementia and their carers: a systematic review and critical discussion. J Pain Symptom Manag.

[5] Wendrich-van Dael A, Bunn F, Lynch J, Pivodic L, Van den Block L, Goodman C (2020). Advance care planning for people living with dementia: an umbrella review of effectiveness and experiences. Int J Nurs Stud.

[6] de Boer ME, Dröes RM, Jonker C, Eefsting JA, Hertogh CM (2010). [The lived-experiences of early-stage dementia and the feared suffering: an explorative survey]. Tijdschr Gerontol Geriatr.

[7] Thompson SC, Cheek PR, Graham MA (1988). The other side of perceived control: disadvantages and negative effects. The Social psychology of Health.

[8] Tilburgs B, Vernooij-Dassen M, Koopmans R, van Gennip H, Engels Y, Perry M (2018). Barriers and facilitators for GPs in dementia advance care planning: a systematic integrative review. PLoS ONE.

[9] Keijzer-van Laarhoven AJ, Touwen DP, Tilburgs B, van Tilborg-den Boeft M, Pees C, Achterberg WP (2020). Which moral barriers and facilitators do physicians encounter in advance care planning conversations about the end of life of persons with dementia? A meta-review of systematic reviews and primary studies. BMJ open.

[10] van Soest-Poortvliet MC, van der Steen JT, Gutschow G, Deliens L, Onwuteaka-Philipsen BD, de Vet HC (2015). Advance care planning in nursing home patients with dementia: a qualitative interview study among family and professional caregivers. J Am Med Dir Assoc.

[11] van der Steen JT, Ribbe MW, Deliens L, Gutschow G, Onwuteaka-Philipsen BD (2014). Retrospective and prospective data collection compared in the dutch end of life in Dementia (DEOLD) study. Alzheimer Disease & Associated Disorders.

[12] Deliens L, Van der Wal G (2003). The euthanasia law in Belgium and the Netherlands. The Lancet.

[13] Chan B, Somerville M (2016). Converting the ‘right to life’to the ‘right to physician-assisted suicide and euthanasia’: an analysis of Carter v Canada (Attorney General), Supreme Court of Canada. Med Law Rev.

[14] Québec Official Publisher. Quebec National Assembly, Bill 52: An act respecting end-of-life care. http://www2.publicationsduquebec.gouv.qc.ca/dynamicSearch/telecharge.php?type=5&file=2014C2F.PDF2014 Accessed July 19, 2023.

[15] World Federation Right to die Societies. Legal situation https://wfrtds.org/worldmap/germany/2022 Accessed July 19, 2023.

[16] Radbruch L, Leget C, Bahr P, Müller-Busch C, Ellershaw J, de Conno F (2016). Euthanasia and physician-assisted suicide: a white paper from the European Association for Palliative Care. Palliat Med.

[17] Inglehart RC, Nash R, Hassan QN, Schwartzbaum J. Attitudes toward Euthanasia: a longitudinal analysis of the role of Economic, Cultural, and Health-Related factors. Journal of Pain and Symptom Management; 2021. 10.1016/j.jpainsymman.2021.01.009 33493587

[18] Cohen J, Van Landeghem P, Carpentier N, Deliens L (2014). Public acceptance of euthanasia in Europe: a survey study in 47 countries. Int J Public Health.

[19] Emanuel EJ, Onwuteaka-Philipsen BD, Urwin JW, Cohen J (2016). Attitudes and practices of euthanasia and physician-assisted suicide in the United States, Canada, and Europe. JAMA.

[20] Brinkman-Stoppelenburg A, Evenblij K, Pasman HRW, van Delden JJ, Onwuteaka‐Philipsen BD, van der Heide A (2020). Physicians’ and public attitudes toward euthanasia in people with advanced dementia. J Am Geriatr Soc.

[21] Okishiro N, Miyashita M, Tsuneto S, Sato K, Shima Y (2009). The Japan HOspice and Palliative Care evaluation study (J-HOPE study): views about legalization of death with dignity and euthanasia among the bereaved whose family member died at palliative care units. Am J Hospice Palliat Medicine®.

[22] Tanida N (2000). The view of religions toward euthanasia and extraordinary treatments in Japan. J Relig Health.

[23] Takeo K, Satoh K, Minamisawa H, Mitoh T (1991). Health workers’ attitudes toward euthanasia in Japan. Int Nurs Rev.

[24] Asai A, Ohnishi M, Nagata SK, Tanida N, Yamazaki Y (2001). Doctors’ and nurses’ attitudes towards and experiences of voluntary euthanasia: survey of members of the Japanese Association of Palliative Medicine. J Med Ethics.

[25] Mitchell SL, Kiely DK, Hamel MB (2004). Dying with advanced dementia in the nursing home. Arch Intern Med.

[26] Kaasa S, Loge JH, Fayers P, Caraceni A, Strasser F, Hjermstad MJ (2008). Symptom assessment in palliative care: a need for international collaboration. J Clin Oncol.

[27] Kunz M, Seuss D, Hassan T, Garbas JU, Siebers M, Schmid U (2017). Problems of video-based pain detection in patients with dementia: a road map to an interdisciplinary solution. BMC Geriatr.

[28] Werner P, Al-Hamadi A, Niese R, Walter S, Gruss S, Traue HC, editors. Automatic pain recognition from video and biomedical signals. 22nd International Conference on Pattern Recognition; 2014: IEEE.

[29] Taylor SE, Helgeson VS, Reed GM, Skokan LA (1991). Self-generated feelings of Control and Adjustment to Physical Illness. J Soc Issues.

[30] Taylor SE, Armor DA (1996). Positive illusions and coping with adversity. J Pers.

[31] Evers AW, Kraaimaat FW, van Lankveld W, Jongen PJ, Jacobs JW, Bijlsma JW (2001). Beyond unfavorable thinking: the illness cognition questionnaire for chronic diseases. J Consult Clin Psychol.

[32] Brandtstädter J, Rothermund K (1994). Self-percepts of control in middle and later adulthood: buffering losses by rescaling goals. Psychol Aging.

[33] Antonovsky H, Sagy S (1986). The development of a sense of coherence and its impact on responses to stress situations. J Soc Psychol.

[34] Antonovsky A. The life cycle, mental health and the sense of coherence. Isr J Psychiatry Relat Sci. 1985. 3836223

[35] Ruhnke GW, Wilson SR, Akamatsu T, Kinoue T, Takashima Y, Goldstein MK (2000). Ethical decision making and patient autonomy: a comparison of physicians and patients in Japan and the United States. Chest.

[36] van der Steen JT. Dying with dementia: what we know after more than a decade of research. J Alzheimers Disease. 2010;22. 10.3233/JAD-2010-100744 20847433

[37] van der Steen JT, Onwuteaka-Philipsen BD, Knol DL, Ribbe MW, Deliens L (2013). Caregivers’ understanding of dementia predicts patients’ comfort at death: a prospective observational study. BMC Med.

[38] Mitchell SL, Mor V, Gozalo PL, Servadio JL, Teno JM (2016). Tube feeding in US nursing home residents with advanced dementia, 2000–2014. JAMA.

[39] Nakanishi M, Hattori K (2014). Percutaneous endoscopic gastrostomy (PEG) tubes are placed in elderly adults in Japan with advanced dementia regardless of expectation of improvement in quality of life. J Nutr Health Aging.

[40] Kouwenhoven PS, Raijmakers NJ, van Delden JJ, Rietjens JA, Van Tol DG, van de Vathorst S (2015). Opinions about euthanasia and advanced dementia: a qualitative study among dutch physicians and members of the general public. BMC Med Ethics.

[41] Weyers H (2006). Explaining the emergence of euthanasia law in the Netherlands: how the sociology of law can help the sociology of bioethics. Sociol Health Illn.

[42] Helton MR, van der Steen JT, Daaleman TP, Gamble GR, Ribbe MW (2006). A cross-cultural study of physician treatment decisions for demented nursing home patients who develop pneumonia. The Annals of Family Medicine.

[43] Buiting HM, van Delden JJ, Rietjens JA, Onwuteaka-Philipsen BD, Bilsen J, Fischer S (2007). Forgoing artificial nutrition or hydration in patients nearing death in six european countries. J Pain Symptom Manag.

[44] van der Steen JT, Hertogh CM, de Graas T, Nakanishi M, Toscani F, Arcand M (2013). Translation and cross-cultural adaptation of a family booklet on comfort care in dementia: sensitive topics revised before implementation. J Med Ethics.

[45] Gysels M, Evans N, Meñaca A, Andrew E, Toscani F, Finetti S (2012). Culture and end of life care: a scoping exercise in seven european countries. PLoS ONE.

[46] Helton MR, van der Steen JT, Daaleman TP, Gamble GR, Ribbe MW. A cross-cultural study of physician treatment decisions for demented nursing home patients who develop pneumonia. Ann Fam Med. 2006;4. 10.1370/afm.536 PMC1479435 16735523

[47] Huber M, Knottnerus JA, Green L, Van Der Horst H, Jadad AR, Kromhout D et al. How should we define health? BMJ. 2011;343. 10.1136/bmj.d4163 21791490

[48] Cars O, Mölstad S, Melander A (2001). Variation in antibiotic use in the European Union. The Lancet.

[49] van der Steen JT, Kruse RL, Ooms ME, Ribbe MW, van der Wal G, Heintz LL et al. Treatment of nursing home residents with dementia and lower respiratory tract infection in the United States and the Netherlands: an ocean apart. J Am Geriatr Soc. 2004;52. 10.1111/j.1532-5415.2004.52204.x 15086647

[50] van der Steen JT, Meuleman-Peperkamp I, Ribbe MW. Trends in treatment of pneumonia among dutch nursing home patients with dementia. J Palliat Med. 2009;12. 10.1089/jpm.2009.0049 19622013

[51] The A-M, Pasman R, Onwuteaka-Philipsen B, Ribbe M, van der Wal G (2002). Withholding the artificial administration of fluids and food from elderly patients with dementia: ethnographic study. BMJ.

[52] Löfmark R, Nilstun T, Cartwright C, Fischer S, Van Der Heide A, Mortier F (2008). Physicians’ experiences with end-of-life decision-making: survey in 6 european countries and Australia. BMC Med.

[53] van der Steen JT, Galway K, Carter G, Brazil K (2016). Initiating advance care planning on end-of-life issues in dementia: ambiguity among UK and Dutch physicians. Arch Gerontol Geriatr.

[54] Krause S, Rydall A, Hales S, Rodin G, Lo C (2015). Initial validation of the death and dying distress scale for the assessment of death anxiety in patients with advanced cancer. J Pain Symptom Manag.

[55] Lo C, Hales S, Zimmermann C, Gagliese L, Rydall A, Rodin G (2011). Measuring death-related anxiety in advanced cancer: preliminary psychometrics of the death and dying distress scale. J Pediatr Hematol Oncol.

[56] Clark CM, Ewbank DC (1996). Performance of the dementia severity rating scale: a caregiver questionnaire for rating severity in Alzheimer disease. Alzheimer Dis Assoc Disord.

[57] Hennink M, Kaiser BN (2022). Sample sizes for saturation in qualitative research: a systematic review of empirical tests. Soc Sci Med.

[58] Krause N (2015). Trust in God, forgiveness by God, and death anxiety. OMEGA-Journal of Death and Dying.

[59] Kumabe T (2006). Psychological study of japanese attitudes of life and death-4 factors influencing attitudes to death. Health Psychol Res.

[60] Wong PT, Reker GT, Gesser G (1994). Death attitude Profile-Revised: a multidimensional measure of attitudes toward death. Death anxiety handbook: research, instrumentation, and application.

[61] Higginson IJ, Gao W, Jackson D, Murray J, Harding R (2010). Short-form Zarit Caregiver Burden interviews were valid in advanced conditions. J Clin Epidemiol.

[62] Arai Y, Kei K, Hosokawa T, Washio M, Miura H, Hisamichi S (1997). Reliability and validity of the japanese version of the Zarit Caregiver Burden interview. J Neuropsychiatry Clin Neurosci.

[63] Braun M, Scholz U, Hornung R, Martin M (2010). The burden of spousal caregiving: a preliminary psychometric evaluation of the german version of the Zarit burden interview. Aging Ment Health.

[64] Iecovich E (2012). Psychometric properties of the Hebrew version of the Zarit Caregiver Burden Scale short version. Aging Ment Health.

[65] Smaling HJA, Joling KJ, van de Ven PM, Bosmans JE, Simard J, Volicer L (2018). Effects of the Namaste Care Family programme on quality of life of nursing home residents with advanced dementia and on family caregiving experiences: study protocol of a cluster-randomised controlled trial. BMJ open.

[66] Gaugler JE, Mittelman MS, Hepburn K, Newcomer R (2010). Clinically significant changes in burden and depression among dementia caregivers following nursing home admission. BMC Med.

[67] Steinhauser KE, Clipp EC, Bosworth HB, McNeilly M, Christakis NA, Voils CI (2004). Measuring quality of life at the end of life: validation of the QUAL-E. Palliat Support Care.

[68] Steinhauser KE, Bosworth HB, Clipp EC, McNeilly M, Christakis NA, Parker J (2002). Initial assessment of a new instrument to measure quality of life at the end of life. J Palliat Med.

[69] Carver CS (1997). You want to measure coping but your protocol’s too long: consider the brief cope. Int J Behav Med.

[70] Otsuka Y, Sasaki T, Iwasaki K, Mori I (2009). Working hours, coping skills, and psychological health in japanese daytime workers. Ind Health.

[71] van der Steen JT, Arcand M, Toscani F, de Graas T, Finetti S, Beaulieu M (2012). A family booklet about comfort care in advanced dementia: three-country evaluation. J Am Med Dir Assoc.

[72] Galvin JE (2015). The Quick Dementia Rating System (QDRS): a rapid dementia staging tool. Alzheimer’s & dementia: diagnosis. Assess Disease Monit.

[73] Annear MJ, Otani J, Li J (2017). Japanese-language Dementia Knowledge Assessment Scale: psychometric performance, and health student and professional understanding. Geriatr Gerontol Int.

[74] Annear MJ, Toye C, Elliott K-EJ, McInerney F, Eccleston C, Robinson A (2017). Dementia knowledge assessment scale (DKAS): confirmatory factor analysis and comparative subscale scores among an international cohort. BMC Geriatr.

[75] Brazil K, Carter G, Galway K, Watson M, van der Steen JT (2015). General practitioners perceptions on advance care planning for patients living with dementia. BMC Palliat care.

[76] de Raaij EJ, Schröder C, Maissan FJ, Pool JJ, Wittink H (2012). Cross-cultural adaptation and measurement properties of the brief illness perception questionnaire-dutch language version. Man Therap.

[77] Broadbent E, Petrie KJ, Main J, Weinman J (2006). The brief illness perception questionnaire. J Psychosom Res.

[78] Valenta S, De Geest S, Fierz K, Beckmann S, Halter J, Schanz U (2017). Perception of late effects among long-term survivors after haematopoietic stem cell transplantation: descriptive analysis and validation of the brief illness perception Questionnaire. A sub-study of the PROVIVO study. Eur J Oncol Nurs.

[79] Aron A, Aron EN, Smollan D (1992). Inclusion of other in the self scale and the structure of interpersonal closeness. J Personal Soc Psychol.

[80] Kovaleva A. The IE-4: construction and validation of a short scale for the assessment of locus of control. DEU; 2012.

[81] Kroenke K, Spitzer RL, Williams JB (2003). The Patient Health Questionnaire-2: validity of a two-item depression screener. Med Care.

[82] van der Steen JT, Toscani F, de Graas T, Finetti S, Nakanishi M, Nakashima T (2011). Physicians’ and nurses’ perceived usefulness and acceptability of a family information booklet about comfort care in advanced dementia. J Palliat Med.

[83] Schulz R, Boerner K, Klinger J, Rosen J (2015). Preparedness for death and adjustment to bereavement among caregivers of recently placed nursing home residents. J Palliat Med.

[84] Durepos P, Akhtar-Danesh N, Ploeg J, Sussman T, Kaasalainen S. Caring ahead: mixed methods development of a questionnaire to measure caregiver preparedness for end-of-life with dementia. Palliat Med. 2021:0269216321994732. 10.1177/0269216321994732 PMC8022086 33619975

[85] van der Steen JT, Azizi B, Nakanishi M, Shinan-Altman S, Mehr DR, Radbruch L (2020). Cross‐cultural acceptability of interventions at the end of life in dementia: video vignette study design and pilot evaluation (ERC CONT‐END WP1) Dementia—Cross‐cultural investigations. Alzheimer’s Dement.

